# Moderate-to-vigorous physical activity attenuates the detrimental effects of television viewing on the cardiorespiratory fitness in Asian adolescents: the Asia-fit study

**DOI:** 10.1186/s12889-019-8079-0

**Published:** 2019-12-27

**Authors:** Tetsuhiro Kidokoro, Koya Suzuki, Hisashi Naito, Govindasamy Balasekaran, Jong Kook Song, Soo Yeon Park, Yiing Mei Liou, Dajiang Lu, Bee Koon Poh, Kallaya Kijboonchoo, Stanley Sai-chuen Hui

**Affiliations:** 1 Department of Sports Science, Juntendo University, 1-1 Hiraka-gakuendai, Inzai, Chiba, 270-1695 Japan; 2 Department of Health & Physical Education, International Christian University, 3-10-2 Osawa, Mitaka, Tokyo, 181-8585 Japan; 3 Physical Education & Sports Science, Nanyang Technological University, NIE5-03-37, 1 Nanyang Walk, Singapore, 637616 Singapore; 4 Graduate School of Physical Education, Kyung Hee University, 1732, Deogyeong-daero, Giheung-gu, Yongin-si, Gyeonggi-do 17104 South Korea; 5 Graduate School of Education, Yong In University, 134 Cheoin-gu, Gyeonggi-do, Yongin, 17104 South Korea; 6 School of Nursing, National Yang-Ming University, 155, Sec. 2, Linong Street, Taipei, 112 Taiwan; 7 School of Kinesiology, Shanghai University of Sport, 399 Chang Hai Road, Shanghai, 200438 China; 8 Faculty of Health Sciences, Universiti Kebangsaan Malaysia, 43600 UKM, Bangi Selangor, Kuala Lumpur, Malaysia; 9 Institute of Nutrition, Mahidol University, 999 Phuttamonthon, 4 Road, Salaya, Nakhon Pathom, 73170 Thailand; 10 Department of Sports Science and Physical Education, The Chinese University of Hong Kong, Rm G10, Kwok Sports Building, Shatin, Hong Kong

**Keywords:** Exercise, Sitting time, Aerobic fitness, Asian, Adolescents

## Abstract

**Background:**

Moderate-to-vigorous physical activity (MVPA) and television viewing are independently associated with cardiorespiratory fitness. However, limited evidence is available on their combined effects, specifically of MVPA and watching television, on cardiorespiratory fitness in the young Asian population. Therefore, the present study examined whether MVPA can attenuate the detrimental effects of prolonged television viewing on the cardiorespiratory fitness of Asian adolescents.

**Methods:**

This is a cross-sectional study on 9553 adolescents (aged 12–15 years) from 8 Asian metropolitan cities (Tokyo, Hong Kong, Shanghai, Taipei, Bangkok, Kuala Lumpur, Seoul, and Singapore). Cardiorespiratory fitness was assessed by using a 15-m progressive aerobic capacity endurance run (PACER) test. The time spent on MVPA and watching television was assessed using the International Physical Activity Questionnaire-Short Form.

**Results:**

MVPA was more closely associated with the PACER score than the duration of watching television. Compared with the reference group (i.e. those with the lowest levels of MVPA [< 30 min/day] and the most sedentary [≥3 h/day of television time]), PACER scores were significantly higher for those who met the physical activity recommendation (≥60 min/day in MVPA), regardless of the duration of television viewing. Conversely, girls in the least active group (< 30 min/day of MVPA) who watched television < 1 h/day demonstrated better PACER scores than the reference group.

**Conclusions:**

Sufficient MVPA (≥60 min/day) can attenuate the detrimental effects of excessive television viewing with cardiorespiratory fitness in Asian adolescents. In addition, the duration of television viewing had significant but weaker associations with cardiorespiratory fitness compared to MVPA.

## Background

Cardiorespiratory fitness (CRF) reflects the overall capacity of the cardiovascular and pulmonary systems to supply oxygen during sustained exercise, as well as the ability to perform such exercise [1]. There is growing evidence of an inverse relationship of CRF with various important health markers in adolescents [2, 3]. In addition, longitudinal studies suggest that higher CRF during adolescence is associated with a healthier cardiovascular profile [4] and a healthier body composition [5] later in life. Furthermore, CRF can be tracked well from childhood to adulthood [6]. Therefore, improving CRF at early ages is imperative for lifetime health promotion.

CRF is influenced by several factors including genetics, yet its principal modifiable determinants are physical activity and sedentary behaviour [7]. Evidence has shown that moderate-to-vigorous physical activity (MVPA) is favourably associated with better CRF [8]. In addition, growing evidence indicates that increased sedentary behaviour, such as watching television daily, is associated with low CRF [9]. Importantly, there is often little association between MVPA and television viewing [10, 11], suggesting that MVPA and television viewing are independently associated with CRF.

Despite the above evidence, a previous study suggested that physical activity might be more closely associated with health outcomes than the amount of total sedentary behaviour [12]. Here, it was suggested that sufficient physical activity (60–75 min/day) can attenuate the detrimental influence of prolonged sedentary time on all-cause mortality in adults [12]. This is an important implication because in recent societies, long sitting periods might be unavoidable in many situations. For example, school-aged adolescents spend most of their awake time sitting in classrooms [13, 14]. If sufficient physical activity can attenuate the effects of prolonged sitting among adolescents, promoting physical activity can potentially combat a sedentary society.

Previous studies examined the independent and combined effects of MVPA and sedentary behaviour on the CRF of adolescents, and achieved inconclusive results [15–18]. For example, Santos et al. reported that MVPA and sedentary behaviours were independently associated with CRF [15], while Bai et al. demonstrated that only MVPA, not sedentary time, was associated with CRF [16]. Importantly, most studies investigating the effect of physical activity and sedentary behaviour on CRF were performed among adolescents residing in western countries, with only a few studies on non-white populations, including Asian adolescents. This is a key limitation in the literature because there seems to be ethnic differences in the effects of physical activity on CRF between European and South Asian adults [19]. Furthermore, evidence shows that CRF in Asian adolescents substantially declined in recent decades [20], which warrants prompt actions among these populations. Increased understanding of the combined effects of MVPA and television viewing will help to inform public health messages and policies aimed at improving the CRF of Asian adolescents.

Therefore, the purpose of the present study was to examine the combined effects of MVPA and television viewing on the CRF of Asian adolescents. Based on a previous study conducted on healthy adults [12], it was hypothesised that a high level of MVPA will attenuate the detrimental effects of excessive television viewing with CRF on Asian adolescents.

## Methods

### Study design and sampling

The Asia-Fit Study is a cross-sectional study that simultaneously investigates the associations of multiple lifestyle behaviours with physical fitness among Asian adolescents. The target group was adolescents aged 12 to 15 years, living in eight Asian metropolitan cities (Tokyo, Hong Kong, Shanghai, Taipei, Bangkok, Kuala Lumpur, Seoul, and Singapore). Participants were recruited from each city using stratified sampling procedures based on the geographic regions, school districts, school type, and gender distribution of each city. Specifically, one or more school districts were selected within each city. The primary sample unit was schools in which classes that best corresponded to the target age group were considered as the secondary sampling unit. Schools that declined participation were systematically replaced by other randomly selected schools from the same district. According to the power analysis for sample size, our pilot study comparing fitness of Taiwan and Hong Kong adolescents, using β of 0.8 and α of 0.05, showed that the mean ± standard deviation of the coefficient of variation of CRF was 30.06 ± 13.78, and the required sample size was 225 per sex (boys and girls)-age (12–13 years and 14–15 years) group per city. To manage the anticipated possibilities of losing data in a large-scale study, we aimed to recruit approximately 400 participants per sex-age group per city (i.e., 1600 participants from each city in total).

To standardise data collection methodology for each country, multiple research meetings were held including face-to-face meetings and email-based meetings. More specifically, after the research concept was developed among Asia-Fit study research group members through email and video meetings, the project leader (SSH, Hong Kong) of the Asia-Fit study visited the seven other participating cities (Tokyo, Shanghai, Taipei, Bangkok, Kuala Lumpur, Seoul, and Singapore) to explain the research protocol and provide training sessions to the researchers to standardise the method for measuring the outcomes (CRF, questionnaire assessments, and anthropometric assessments).

The outcome assessments were conducted in each country between June 2013 and December 2014. The standard version of the questionnaire in English was translated into the specific language for each country, and back translation was performed to validate the translation.

The whole Asia Fit Study project was conducted in accordance with the Declaration of Helsinki and approved by an institutional review board in the hosting country, Hong Kong (The Chinese University of Hong Kong [CRE-2010.091]). Thereafter, the study was also approved by each of local institutional review boards from the remaining participating countries, which included those from the Tohoku Gakuin University (Tokyo), Shanghai University of Sport (Shanghai), Taoyuan General Hospital (Taipei), Mahidol University (Bangkok), Universiti Kebangsaan Malaysia (Kuala Lumpur), Kyung Hee University (Seoul), and Nanyang Technological University (Singapore). Written informed consent was obtained from each participant’s parent or legal guardian, after they were provided a complete written explanation of the study including its aims, protocol, and possible occurrence of discomfort and risks. The participants were made aware of their right to withdraw consent for study-participation at any time, without prejudice.

### Assessments of CRF

CRF was assessed by a 15-m progressive aerobic capacity endurance run (PACER) test for measuring aerobic fitness [21]. The participants were asked to run back and forth over a 15-m distance with progressive increases in running pace, which was controlled by pre-recorded pace-music and instruction, until participants were fatigued (i.e. failure to keep up with the running speed twice). Several running laps for practice were provided before the test trial. The maximal number of running laps the participants completed in the test trial was recorded as their performance in aerobic fitness. The pre-recorded PACER music and verbal instructions were translated into different languages relevant to each city. As PACER scores differed among age groups (12 years and 13 years < 14 years and 15 years) and sex (boys > girls), age- and sex-specific z-scores of PACER tests were calculated. A larger value indicated a greater CRF level.

### Assessments of MVPA and television viewing time

MVPA and television viewing durations were assessed by self-report. MVPA in the past week was assessed using the International Physical Activity Questionnaire-Short Form (IPAQ-SF), which was developed as a surveillance instrument to measure multiple domains of physical activity [22, 23]. The questionnaire has widely been used in previous international comparison studies [22, 24], and it has acceptable reliability and validity [22]. The IPAQ-SF asks respondents to report frequency and duration of moderate-intensity physical activity (MPA) and vigorous-intensity physical activity (VPA) performed for at least a 10-min duration per session. Time spent in MPA was calculated as the frequency of MPA multiplied by MPA duration, which was then divided by 7. The same formula was used for the time spent in VPA. Time spent in MVPA was calculated as the sum of the daily MPA and VPA. The duration of watching television was assessed by self-reports of participants based on daily average time they engaged in watching television every day. Thereafter, weekly television watching was estimated by taking a weighted average of daily weekday and weekend activity (i.e. weekly television time = [average daily weekday television time × 5] + [average daily weekend television time × 2]).

### Anthropometry assessments

Body weight was measured using digital weighing scale, accurate to the nearest 0.1 kg, and height was measured using stadiometer, accurate to the nearest 0.1 cm. Body mass index (BMI) was calculated as weight (kg) divided by the square of the height (m^2^). The percentage of body fat was measured on the participants who were in a standing position, wearing light clothing and with an empty bladder, using a bioelectrical impedance analysis (Tanita BC-545, Tanita Corp., Tokyo, Japan).

### Statistical analysis

Descriptive characteristics included means for continuous variables and percentages for categorical variables. We used the analysis of variance for ordinal and interval variables or the chi-square test for categorical variables to compare the difference in anthropometry, MVPA, television time, and CRF among the Asian cities.

Linear regression models were used to examine the independent associations of MVPA (min/day) and television time (min/day) with the PACER z-score after adjustment for age, city, and BMI. Thereafter, we mutually adjusted exposures (MVPA and television time) for each other (i.e. when MVPA was modelled as the main exposure, the analysis was adjusted for television time, and when television time was modelled as the main exposure, the analysis was adjusted for MVPA).

Next, we performed combined analyses of the associations of MVPA and television time with the PACER score. Here, participants were categorised into four groups based on time spent in MVPA (< 30 min/day, 30 to < 60 min/day, 60 to < 90 min/day, and ≥ 90 min/day). In addition, according to the time spent watching television, the participants were categorised into four groups (< 1 h, 1 to < 2 h, 2 to < 3 h, ≥3 h). Thereafter, the participants were cross-tabulated into 16 (4 × 4) groups to directly compare groups with different amounts of MVPA and television time against those who had the least MVPA (< 30 min/day) and those who watched television the most (≥3 h/day; i.e. the reference group). An analysis of covariance was performed to examine the differences among the groups after adjusting for age, city, and BMI. All statistical analyses were performed using IBM SPSS Statistics for Windows, version 24.0 (IBM Corporation, Armonk, NY, USA), and a *p*-value < 0.05 denoted statistical significance. Values were reported as means ± standard deviation unless otherwise stated.

## Results

Among 12,590 adolescents from whom written informed consent was obtained from their parents or guardians, 3037 participants did not provide valid data on height (*n* = 121), weight (*n* = 123), PACER score (*n* = 311), MVPA (*n* = 1909), and/or television time (*n* = 855), therefore, those participants were excluded from the final analysis. The final sample for the present study comprised 9553 Asian adolescents aged 12–15 years (5094 boys and 4459 girls; valid data = 75.8%).

### Basic characteristics

Descriptive characteristics of the participants are shown in Table 1. Significant differences existed in MVPA, television time, and PACER score in adolescents across cities (*p* < 0.001). Adolescents in Tokyo were the most active (MVPA = 73.5 ± 76.4 min/day), and had the best CRF (PACER z-score = 1.11 ± 1.14). Adolescents in Shanghai spent the least amount of time watching television (television time = 1.24 ± 0.99 h/day); however, they were also the least active (MVPA = 37.9 ± 26.3 min/day). The poorest CRF was observed in adolescents from Bangkok (PACER z-score = − 0.53 ± 0.74). The time spent watching television was the greatest in adolescents from Kuala Lumpur (television time = 2.88 ± 1.02 h/day).

**Table 1:** Descriptive characteristics of the participants in the Asian cities

	Tokyo	Hong Kong	Shanghai	Taipei	Bangkok	Kuala Lumpur	Seoul	Singapore	Total	*p* value	Post-hoc tests
Number of subjects	1045	1132	1485	1336	972	1434	1247	1096	9747		
Age (y)	13.3 ± 0.9	13.5 ± 1.0	14.0 ± 0.8	13.8 ± 0.9	14.0 ± 0.9	13.7 ± 1.0	13.4 ± 1.0	13.5 ± 1.2	13.6 ± 1.0	< 0.001	Shanghai, Bangkok > Taipei, Kuala Lumpur > Hong Kong, Singapore > Seoul, Tokyo
% of boys (n)	52.7 (551)	56.2 (636)	51.1 (759)	53.6 (716)	50.0 (486)	49.7 (712)	60.1 (749)	54.4 (596)	53.4 (5205)	0.147	
Anthropometric characteristics											
Height (cm)	157.5 ± 8.1	160.8 ± 8.3	164.4 ± 7.8	160.9 ± 7.9	158.9 ± 8.0	155.6 ± 8.2	161.1 ± 8.2	159.8 ± 8.9	160.0 ± 8.6	< 0.001	Shanghai > Seoul, Taipei, Hong Kong > Singapore, Bangkok > Tokyo > Kuala Lumpur
Weight (kg)	48.8 ± 10.3	52.3 ± 12.5	56.5 ± 12.2	55.6 ± 13.3	53.2 ± 14.4	49.7 ± 12.1	55.6 ± 11.7	52.0 ± 13.4	53.0 ± 12.7	< 0.001	Shanghai, Seoul, Taipei > Bangkok, Hong Kong, Singapore > Kuala Lumpur, Tokyo
Body mass index (kg/m^2^)	19.5 ± 3.1	20.1 ± 3.9	20.8 ± 3.7	21.3 ± 4.3	20.9 ± 4.8	20.4 ± 4.2	21.3 ± 3.6	20.2 ± 4.2	20.6 ± 4.0	< 0.001	Seoul, Taipei, Bangkok, Shanghai, Kuala Lumpur > Singapore, Hong Kong > Tokyo
MVPA (min/day)	73.5 ± 76.4	38.0 ± 53.6	37.9 ± 26.3	43.3 ± 59.4	42.8 ± 56.1	39.9 ± 44.7	59.7 ± 69.2	52.2 ± 58.5	47.9 ± 57.7	< 0.001	Tokyo > Seoul > Singapore > Taipei, Bangkok, Kuala Lumpur, Hong Kong, Shanghai
Television time (h/day)	2.50 ± 1.45	1.69 ± 1.36	1.24 ± 0.99	1.80 ± 1.31	1.81 ± 1.34	2.88 ± 1.02	1.49 ± 1.21	1.56 ± 1.34	1.87 ± 1.36	< 0.001	Kuala Lumpur > Tokyo > Bangkok, Taipei, Hong Kong, Singapore, Seoul > Shanghai
15-m PACER z-score	1.11 ± 1.14	− 0.24 ± 0.83	0.04 ± 0.76	− 0.13 ± 0.85	−0.53 ± 0.74	−0.43 ± 0.74	−0.01 ± 0.83	0.00 ± 1.06	0.00 ± 1.00	< 0.001	Tokyo > Shanghai, Singapore, Seoul > Taipei > Hong Kong > Kuala Lumpur, Bangkok

Data are presented as a mean ± standard deviation or percentage. *MVPA* moderate-to-vigorous physical activity, *PACER* progressive aerobic capacity endurance run test

### Independent associations of MVPA and television time with PACER

Table 2 shows independent associations of MVPA and television time with PACER z-scores. MVPA was positively associated with PACER z-scores for both sexes (boys: β = 0.322, *p* < 0.001; girls: β = 0.256, *p* < 0.001) after adjustment for age, city, and BMI (Model 1). The results remained significant even after further adjustment for television time for both sexes (boys: β = 0.323, *p* < 0.001; girls: β = 0.253, *p* < 0.001) (Model 2). In contrast, television time was negatively associated with PACER z-scores only among girls (β = − 0.072, *p* < 0.001). The results remained significant even after adjusting further for MVPA among girls (β = − 0.072, *p* < 0.001). There was no significant association between television time and the PACER z-score among boys (*p* > 0.363).

**Table 2:** Independent associations of MVPA and television time with PACER z-score

	B	SEB	β	p
Boys				
Model 1				
MVPA (min/day)	0.005	0.001	0.322	< 0.001
Television time (h/day)	−0.006	0.009	−0.008	0.540
Model 2				
MVPA (min/day)	0.005	0.001	0.323	< 0.001
Television time (h/day)	−0.008	0.009	−0.011	0.363
Girls				
Model 1				
MVPA (min/day)	0.006	0.001	0.254	< 0.001
Television time (h/day)	−0.053	0.010	−0.072	< 0.001
Model 2				
MVPA (min/day)	0.006	0.001	0.253	< 0.001
Television time (h/day)	−0.049	0.010	−0.072	< 0.001

Model 1: Adjusted for age, city, and body mass index

Model 2: Adjusted for model 1 + MVPA for television time or television time for MVPA

*MVPA* moderate-to-vigorous physical activity, *PACER* progressive aerobic capacity endurance run test

### Joint associations of MVPA and television time with PACER

**Fig. 1:**
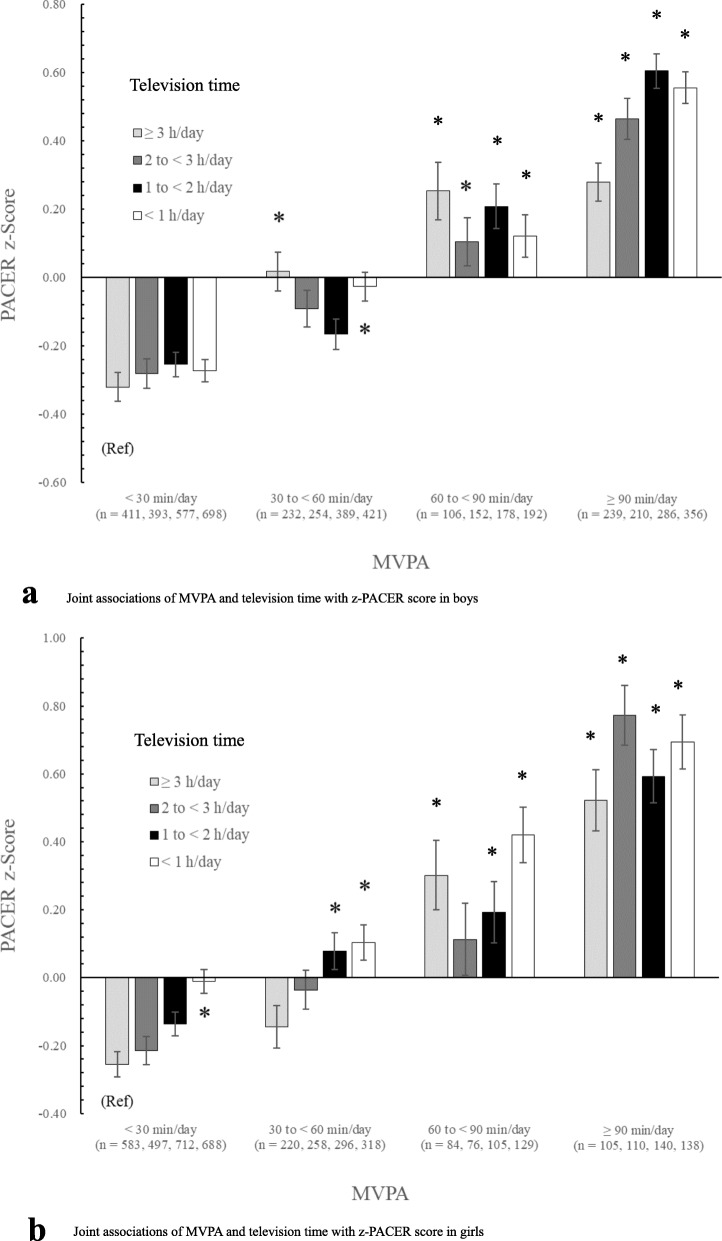
**a** Joint associations of MVPA and television time with the PACER z-score in boys. Data are presented as the mean ± standard error. Analysis of covariance was performed to examine the differences among the groups after adjusting for age, city, and body mass index (F _(18, 5075)_ = 41.6, *p* < 0.001). The reference categories (Ref) are the groups with the lowest levels of MVPA (< 30 min/day) in combination with the most sedentary (≥3 h/day of television time). * Significantly different from Ref. MVPA, moderate-to-vigorous physical activity; PACER, progressive aerobic capacity endurance run test. **b** Joint associations of MVPA and television time with the PACER z-score in girls. Data are presented as a mean ± standard error. Analysis of covariance was performed to examine the differences among the groups after adjusting for age, city, and body mass index (F _(18, 4440)_ = 25.5, *p* < 0.001). The reference categories (Ref) are the groups with the lowest levels of MVPA (< 30 min/day) in combination with the most sedentary (≥3 h/day of television time). * Significantly different from Ref. MVPA: moderate-to-vigorous physical activity; PACER: progressive aerobic capacity endurance run test

The joint associations of time spent in MVPA and television time with PACER z-scores are shown in Fig. 1a and b. Compared with the reference group (i.e. those who had MVPA < 30 min/day and watched television > 3 h/day), participants with MVPA > 60 min/day demonstrated a significantly better PACER z-score for both sexes, regardless of television time (*p* < 0.05). For girls, those in the second least active group (30 to < 60 min/day of MVPA) showed a significantly better PACER z-score than the reference group, except for girls watching television > 2 h/day. In addition, girls in the least active group (< 30 min/day of MVPA) and watching television < 1 h/day demonstrated better PACER z-scores than the reference group (Fig. 1a/b).

## Discussion

The present study showed that MVPA was more closely associated with CRF than television time among Asian adolescents. The main finding in our study indicates that higher durations of MVPA (≥ 60 min/day) can attenuate the detrimental effects of sedentary activities with CRF for Asian adolescents. Regardless of the time spent watching television, those in the active categories (MVPA 60 to < 90 min/day and ≥ 90 min/day) demonstrated significantly better PACER scores than the reference group (i.e. those who had the least MVPA [< 30 min/day] and watched television the most [≥3 h/day]). In addition, television time had significant but weaker associations with CRF compared to MVPA. However, reducing television time can still be beneficial for the least active girls. Indeed, our joint analyses (MVPA × television time) showed that girls in the second least active group (30 to < 60 min/day of MVPA) showed significantly better PACER scores than the reference group; however, girls who watched television > 2 h/day did not. Additionally, girls in the least active group (< 30 min/day of MVPA) who watched television < 1 h/day demonstrated better PACER scores than the reference group.

Our findings from the joint analysis demonstrated that if sufficient MVPA is accumulated, the detrimental influences of television time can be attenuated regardless of time spent watching television. The level of MVPA required to attenuate the negative effects of television viewing was ≥60 min/day of MVPA, which is congruent with the international youth physical activity guidelines [25]. Previous studies demonstrated that accumulating at least 60 min/day of MVPA can enhance CRF, cardiometabolic biomarkers, body composition, and mental health, among other health benefits [8, 26]. Recently, growing evidence shows that prolonged sedentary behaviour is associated with poor CRF [9]. Despite the robust evidence regarding sedentary behaviour, in a real society, breaking up prolonged sitting time can be challenging. School-aged adolescents, in particular, are required to sit still for prolonged periods on many occasions (e.g. in a school setting or for academic requirements). The present study suggests that if prolonged sedentary behaviour is unavoidable, performing greater durations of MVPA is important for improving CRF.

Although MVPA appears to be more important than time spent watching television in relation to CRF among Asian adolescents, the present study also suggests that reducing daily television time may still be beneficial for improving CRF, at least for physically inactive girls (MVPA < 60 min/day). This has important implications because girls are usually less active than boys, and most girls do not meet physical activity recommendations [27]. Indeed, only 20.4% of girls in our sample cohort achieved the recommended physical activity levels while 34.2% of boys met the recommended levels (MVPA ≥60 min/day). It is possible that targeting 60 min/day of MVPA in the first place may be quite intimidating, particularly for adolescents who are otherwise inactive [26], and having an apparently out-of-reach target may undermine the chances of physical activity participation [28]. From this perspective, decreasing television time might be more attainable as an initial goal for increasing CRF for inactive girls.

The present study has many strengths. It is the first study to examine the combinatory effects of MVPA and television time on CRF in Asian adolescents. The present study had a large sample size (*n* = 9553), which enabled us to detect differences in the PACER score among the groups by our joint analyses (MVPA × television time). Moreover, studies investigating the effect of MVPA and sedentary lifestyle on CRF were performed among adolescents in western countries, with only a few studies on non-white populations, including Asian adolescents. In particular, further evidence is required for Asian adolescents, since their CRF has substantially declined [20]. The present study reveals the combinatory effects of MVPA and television time on Asian adolescents, which can inform public health organisations and policymakers aimed at improving the CRF of those populations.

Despite the insights provided in our study, some limitations need to be considered. First, it was not possible to infer causal relationships between MVPA, television time, and CRF because a cross-sectional design was used. Indeed, it is possible that physically fit adolescents are more likely to participate in MVPA (or reduced sedentary behaviour) as bi-directional associations among those outcomes have been reported previously [29, 30]. Second, MVPA and television time were assessed by self-report, which may be lacking in precision. In particular, IPAQ was used to evaluate MVPA in the present study which is recommended for participants of 15 years or older [22]. The validity and reliability of IPAQ for younger adolescents are uncertain. Indeed, potential underestimation in MVPA was reported, in particular, for active adolescents [31]. We suggest that more precise instruments, such as an accelerometer, be used for future studies. Third, our measure of sedentary time was daily television viewing time only instead of total sedentary time; therefore, it is not clear whether sufficient MVPA can still attenuate the detrimental influences of overall sedentary time in Asian adolescents. Indeed, new sedentary activities associated with smartphones, tablets, and other small-screen electronic products have become readily available, and these may impact health outcomes differently [9]. However, it has been suggested that the duration of television viewing negatively influences health outcomes more than total sedentary time [9] as it is often accompanied by snacking and/or soft drink consumption [32, 33]. Fourth, in the present study, we did not evaluate sedentary patterns (e.g. the frequency of breaks during sedentary activities), which is associated with CRF among adolescents [34]. Therefore, the sedentary pattern should be addressed in future studies, particularly for Asian adolescents, as limited evidence is available for the population.

## Conclusions

Sufficient MVPA (≥ 60 min/day) could attenuate the detrimental association of excessive television time with CRF in Asian adolescents. In addition, television time had significant but weaker associations with CRF, compared to MVPA.

## Data Availability

The datasets analysed during the current study are available from the corresponding author on reasonable request.

## Abbreviations

BMI: Body mass index
CRF: Cardiorespiratory fitness
IPAQ-SF: Physical Activity Questionnaire-Short Form
MPA: Moderate-intensity physical activity
MVPA: Moderate-to-vigorous physical activity
PACER: Progressive aerobic capacity endurance run
VPA: Vigorous-intensity physical activity
